# The Book World of Medicine and Science

**Published:** 1893-09-30

**Authors:** 


### The Book World of Medicine and Science.

Medical Electricity. By W. E. Steavenson, M.D., and H. Lewis Jones, M.A., M.D. (London: H. K. Lewis.)

Dr. Lewis Jones in the preface to this admirable book states that the materials which had been collected for the work were handed to him with a request that he would complete it for publication. It was found, however, that much would require to be rewritten, although the plan of the book as originally sketched by Dr. Steavenson has been carried out. A large portion of the work is taken up with the physics of the subject, and necessarily so. We thoroughly agree with Dr. Jones that "without a thorough grounding on the physical part of the subject no satisfactory advances can be made in a field of therapeutics which is at present almost entirely neglected by medical men." The chapters devoted to physics are obviously written by one who is a thorough master of his subject, but we would recommend anyone who wishes to do justice to this work to read in the first place an elementary treatise on electricity. The large majority of practitioners know absolutely nothing of electrical physics, and although this may in succeeding years be altered by the greater amount of attention given to the subject by the present generation of medical students, yet we feel that the subject matter is such that a tyro would fail to understand it unless the elements of the subject were first studied.

We question, however, if general practitioners require for the using of electricity as a therapeutic agent such advanced knowledge as is given in the work.

Chapter V., on batteries and apparatus, is clearly and concisely written, and the illustrations given are beautifully executed. The remarks on care of batteries in this chapter are worthy of attention, giving as they do particulars as to the various batteries used.

Chapter VI., on accessory apparatus, is one which we doubt not will be much appreciated by those using electrical batteries, and we would specially notice the remarks on current-collectors, regulation of current, and galvanometers.

The chapter devoted to physiology gives an admirable synopsis of our knowledge of this branch of medicine.

In Chapter VIII. on diagnosis, the author gives very clear instruction as to the methods of procedure with patients who require electrical treatment, and it is here that the knowledge of the advanced physics of the subject is of greater necessity than in dealing with the general therapeutics.

In Chapter IX. Dr. Jones says that in treatment of disease by electricity the following are the first questions to answer: (1) What results are to be expected from the treatment? (2) When should the constant current be used, and when the interrupted? (3) What is the proper strength of current, and the proper duration of treatment? (4) What is to be the direction of the current, and which pole is to be applied to the affected part? (5) What are the manipulations required? To each of these questions the author gives such clear and concise answers, that students of the subject will be able to grasp them without much difficulty, and to apply the knowledge to such cases as they find suitable for the form of treatment. We would have been glad to see a little more space devoted to the condemnation of the so-called electric belts, although

THE BOOK WORLD OF MEDICINE AND SCIENCE—*continued.*

what is said is very much to the point. In a case lately decided, much stronger evidence was given by leading electricians as to the uselessness of these articles than we find here. The three chapters on diseases of the nervous system are carefully and clearly written, and will prove, no doubt, useful to the general practitioner. We are glad to see that Dr. Jones gives particular attention to his authorities, and these will prove of great value to other workers in this subject. The treatment of sciatica and lumbago might have had a little more space devoted to them, considering the frequency of these diseases among all classes. We cannot, however, agree with the author as to the success of electrical treatment in this disease. Our own experience is that very few cases of sciatica are cured by it. Some are temporarily relieved, no doubt, but complete cure has been the exception.

We have nothing but the highest praise for the chapter on electrolysis, the remarks on removal of hairs, naevus, and stricture being thoroughly readable and to the point.

The book is all through very well written, and we feel assured that there is no one, be he general practitioner or specialist, but will benefit largely by its careful perusal. It is by far the best of the books on electricity that we have seen, and ought to be in every medical man's library.

Mountaineering. By Claude Wilson, M.D. The All England Series. (George Bell and Sons.)

Judging by appearance, mountaineering is well thought of by the medical profession since the two most recent works on the subject claim its members as authors.

Although the later book by Dr. Wilson is of modest dimensions compared with its predecessor in the Badminton Library, edited by Mr. Dent, yet its conciseness, eminently practical character, and moderate cost make it a valuable contribution to the literature of the subject. The work is arranged on much the same plan as the Badminton Manual, special chapters being devoted to each branch of the craft, while, in addition, useful hints are given as to guides and guide books, and a chapter deals with the minor bodily ills sometimes experienced by mountain climbers.

The chapter to which, perhaps, most exception can be taken is the introductory, containing the somewhat stereotyped apology for the pursuit, and a justification "that there is an indescribable charm in climbing which appeals to certain natures," which can scarcely be considered either adequate or satisfactory. After all, the "Mountain Gymnast," so forcibly described by Mr. Conway, is somewhat of a rara avis, and his attentions to the mountains are often transitory, while a large number of those to whom a yearly holiday in the mountains has become a habit find their pleasure not in the craft alone, but also in its various associations. It is to these that the average mountaineer can point when interrogated by the sceptic. The special charm of scaling the lofty peak is composite, and only to be really understood by those who have experienced it, while the collateral pleasures are numerous, and to be appreciated by all.

Although a well-worn theme, the special beauties of Nature revealed must be first mentioned. Even the most strenuous decrier of mountaineering has enjoyed a view from a hill, and to outlook alone, in the mountains must be added atmo-

### THE BOOK WORLD OF MEDICINE AND SCIENCE—*(continued)*.

spheric effects not dreamed of by the walker on the plains, but the ordinary experience of the hillman. Then, as an incentive to travelling, how many a piece of country is traversed by the mountaineer, which would have remained for ever unknown to a less adventurous being. Again, perhaps, there is no pursuit so well calculated to bring man and man together and to cultivate an acquaintanceship free and without restraint. What mountaineer does not look back with pleasure on the friendships which the mountains have been the means of initiating and cementing? As a form of sport, perhaps, none brings out more markedly the strong and weak points in a man's character, and so well exhibits the individual in his true colours. The petty hardships of moderate expeditions encourage a fellow-feeling and openness of mind which no other pursuit, except the more serious one of foreign exploration, is likely to do, and as a practice of mental discipline it cannot be valued too highly; small jealousies and personal vanity are necessarily banished from a mountain party, since all must co-operate for the common end, and there is no place for personal display or selfishness.

The question of physical powers is well dealt with by Dr. Wilson, and, given the requisite degree of strength, mountaineering offers certain distinct advantages of its own. The possibility of training by degrees in a short time will suit it to individuals of laborous occupation, while the change of living is a mental tonic comparable perhaps to the physical stimulus and refreshment furnished by the cold bath to a strong man. The freedom of costume and manners, the change of habit involved in the night in the mountain hut or the early start, the regulated exercise, the frequent and irregular meal, the delicious repose after a big expedition, the "off-day" of luxurious idleness, and the fearless disregard of the discomforts dependent on meteorological conditions, all combine to make a mountaineering holiday not a change alone, but an interval in which body and mind are really built up afresh.

The question of "dangers" must, however, be faced, and Dr. Wilson meets it fairly, at the same time clearly showing how those not directly due to atmospheric conditions may be avoided by attention to definite rules, no more rigid or exacting than those governing any other of the higher forms of sport. Here, we trust, the lessons inculcated by our author may be taken to heart, adhesion to such rules as he lays down, easy enough to be guided by, without doubt would have prevented the lamentable accidents chronicled this season. Such a book is particularly well timed, considering the enormous increase in the number of travellers who are now drawn into indulging in a few days' mountaineering by force of their surroundings, and from whom, moreover, the ranks of the mountaineers are mainly increased.

The various chapters deal fully with the several branches of the craft, and bear the stamp of the experienced mountaineer, while not the least useful feature of the book are the lists of desiderata for proper equipment.

### SEPTEMBER REVIEWS.

The New Review is noteworthy, principally for a reply contributed by Dr. Welldon, headmaster of Harrow, to the attack on public schools which appeared there a short time back. Admitting the need for still further reforms, Dr. Welldon bases his defence mainly on the wonderful strides in comfort, in educational standard, and in morality which the last twenty years have witnessed in public schools.

